# Human hybridoma and recombinant Pritumumab antibodies for treatment of human solid tumors

**DOI:** 10.1186/2051-1426-2-S3-P149

**Published:** 2014-11-06

**Authors:** Rishab Gupta, Mark Glassy

**Affiliations:** 1 Nascent Biotech, Inc., San Diego, CA, USA

## 

Pritumumab is a natural human IgG1 kappa antibody that has been derived from a B cell isolated from regional draining lymph node of a cervical carcinoma patient. Specificity analysis of the antibody with human tissues showed the target antigen, altered tumor-associated vimentin, to be highly restricted to various epithelial cancers and not normal cells and tissues. In various Phase II clinical trials in Japan 249 patients with brain cancer were treated with the antibody. Overall response rate was between 25-30%, a 9-fold increase over standard care, with several survivors beyond 5-years post-treatment. Patients were on a low dose regimen of 1 mg given twice a week for a course of 24 weeks, suggesting Pritumumab to be safe and effective in patients with brain cancer. However, the yield from human-hybridoma was only about 1 picogram per cell per day. Therefore, there is a pressing need to produce adequate amount of GMP grade Pritumumab in a cost effective manner. The GPEx^® ^technology of Catalent was adapted to develop a Pritumumab-secreting Chinese hamster ovary (CHO) cell line. This involved construction and cloning of heavy and light chain DNAs into an expression vector and transduced into HEK 293 cells that constitutively produce the MLV *gag, pro*, and *pol*. The Pritumumab cell line was made by performing multiple rounds of transduction (multiplicity of > 1000 retrovector particles/cell) of the GPEx^® ^parental cell line (GCHO) with retrovector made from the gene construct developed to express the Pritumumab antibody light and heavy chains. Five independent transductions were performed: two LC and three HC. Limiting dilution method was used to establish cloned cell lines. Top clones were selected based on antibody titer as determined by Protein A HPLC using a generic IgG standard. Single copies of genes were inserted efficiently in unique locations throughout the genome of the CHO cells to obtain genetically stable cell line that expressed high levels of the antibody. The selected clone represented more than a 40-fold increased production of the antibody over the original human-hybridoma. Flow cytometry binding and immunohistochemical analyses demonstrated comparability between the recombinant and the hybridoma forms of the protein. Furthermore, amino acid compositions of the two forms are identical. Thus, high level of specific productivity and titer of the CHO clone will allow the therapeutic antibody program to move forward into large and extended clinical trials of not only brain cancers but also of other types of cancers as well.

